# Fabrication of PLA/PCL/Graphene Nanoplatelet (GNP) Electrically Conductive Circuit Using the Fused Filament Fabrication (FFF) 3D Printing Technique

**DOI:** 10.3390/ma15030762

**Published:** 2022-01-20

**Authors:** Nour-Alhoda Masarra, Marcos Batistella, Jean-Christophe Quantin, Arnaud Regazzi, Monica Francesca Pucci, Roland El Hage, José-Marie Lopez-Cuesta

**Affiliations:** 1 Polymers Composites and Hybrids (PCH), IMT Mines Ales, 30100 Ales, France; Nour-Alhoda.Masarra@mines-ales.fr (N.-A.M.); marcos.batistella@mines-ales.fr (M.B.); 2 LMGC, IMT Mines Ales, Université Montpellier, CNRS, 30100 Ales, France; jean-christophe.quantin@mines-ales.fr (J.-C.Q.); Arnaud.Regazzi@mines-ales.fr (A.R.); Monica.Pucci@mines-ales.fr (M.F.P.); 3 Laboratory of Physical Chemistry of Materials (LCPM), PR2N-EDST, Faculty of Sciences II, Campus Fanar, Lebanese University, Jdeideh P.O. Box 90656, Lebanon; roland_hag@ul.edu.lb

**Keywords:** electrical conductivity, fused filament fabrication, 3D printing, polymer bio nanocomposites, PLA, PCL, GNP

## Abstract

For the purpose of fabricating electrically conductive composites via the fused filament fabrication (FFF) technique whose properties were compared with injection-moulded properties, poly(lactic acid) (PLA) and polycaprolactone (PCL) were mixed with different contents of graphene nanoplatelets (GNP). The wettability, morphological, rheological, thermal, mechanical, and electrical properties of the 3D-printed samples were investigated. The microstructural images showed the selective localization of the GNPs in the PCL nodules that are dispersed in the PLA phase. The electrical resistivity results using the four-probes method revealed that the injection-moulded samples are insulators, whereas the 3D-printed samples featuring the same graphene content are semiconductors. Varying the printing raster angles also exerted an influence on the electrical conductivity results. The electrical percolation threshold was found to be lower than 15 wt.%, whereas the rheological percolation threshold was found to be lower than 10 wt.%. Furthermore, the 20 wt.% and 25 wt.% GNP composites were able to connect an electrical circuit. An increase in the Young’s modulus was shown with the percentage of graphene. As a result, this work exhibited the potential of the FFF technique to fabricate biodegradable electrically conductive PLA-PCL-GNP composites that can be applicable in the electronic domain.

## 1. Introduction

The use of plastics is significantly increasing in all applications [1]. However, biopolymers have recently become of great interest due to the high demand for sustainable materials possessing eco-friendly characteristics. Among the reasons behind the substitution of synthetic plastic materials is the dissemination of non-degradable plastic waste in oceans and aquatic systems and, consequently, the accompanying danger to aquatic and human life, which has become a major concern. Renewable natural resources are now able to replace commonly used oil-based products to synthesize bioplastics, which are often biodegradable through environmentally friendly bioprocesses [2].

Poly(lactic acid) or polylactide (PLA) is an aliphatic thermoplastic polyester that is biobased, biodegradable, biocompatible, and compostable. It is characterized by important mechanical properties, such as Young’s modulus in the 3–4 GPa range and tensile strength in the 60–70 MPa range [3]. However, PLA features some disadvantages, such as lack of ductility (elongation at break <10%); blending it with other rubbery polymers such as polycaprolactone (PCL) can improve its toughness. PCL is a semi-crystalline aliphatic polyester that is biodegradable and biocompatible but oil-derived. Furthermore, due to the different mechanical and thermal properties of both polymers, their mixture can be a material with well-balanced properties [4].

PLA and PCL can be used as polymeric matrices to be mixed with micro and nano carbonaceous fillers of different geometries, such as carbon black (CB) [5,6], single and multiwall carbon nanotubes (CNT) [7,8,9,10], graphite [11,12], and graphene [13,14] for improving their electrical, mechanical, and thermal properties [15]. Graphene or graphite nanoplatelets (GNP) are composed of stacked graphene sheets connected with each other by Van der Waals forces and feature important electrical properties (electron mobility: 2.5 × 10^5^ cm^2^.V^−1^.s^−1^), as well as outstanding thermal (thermal conductivity above 3000 W.m^−1^.K^−1^) and mechanical properties (Young’s modulus: 1 TPa and tensile strength: 130 GPa) [3]. These and other properties allow GNPs to be integrated into a new generation of high-speed and radio frequency logic devices, electronic circuits, sensors, and solar cells [16]. Moreover, when embedded in polymer matrices, they can improve their electrical conductivity and dielectric properties [17] as well as their mechanical properties, thermal stability, thermal conductivity, gas barrier properties, and dimensional stability [16].

The fused filament fabrication (FFF) technique is an interesting 3D printing technique due to its low cost, ease of use, small size, and the possibility to manufacture multi-functional parts [18]. PLA is the most frequently adopted material by most FFF users [19]. Furthermore, among the different technologies used for graphene-based product fabrication, FFF is promising and the development of new conductive polymer nanocomposites using this technique is highly desirable [20].

Few studies have been carried out to investigate the electrical properties of PLA/PCL composites, and none has used the 3D printing technique. Kim et al. [21] designed electrically conductive PLA/PCL/CB composites by inducing CB particle aggregations in the PCL phase. PCL was added in small amounts; therefore, there was no deterioration of the mechanical performance of the composites. The authors therefore obtained an electrical conductivity of 1 S.m^−1^ and they found the electrical percolation threshold below 4 wt.% of PCL. For preparing their composites, they pre-melted the PLA/PCL via an internal mixer and later added the CB. Xu et al. studied the influence of acid-oxidized multi-wall carbon nanotubes (A-MWCNTs) on the phase morphology and electrical conductivity of PLA/PCL/A-MWCNT composites prepared by micro compounding and featuring PCL percentages ranging from 5 wt.% to 90 wt.%. They realized that the A-MWCNTs were homogeneously and selectively localized in the PCL phase [22]. Huang et al. controlled the localization of MWCNTs at the continuous interface of immiscible PLA/PCL (50 wt.%/50 wt.%) blends through the migration of the MWCNTs from the unfavorable PLA phase to the favorable PCL phase. Because of the accumulation of MWCNTs at the interface, they obtained a very low electrical percolation threshold of 0.025 wt.%. They prepared their composites by the internal mixer to compound the PLA and the MWCNTs together first and to mix the PCL with the PLA/MWCNTs later [23]. It is important to mention that in each of these works, the compression moulding technique was used after melt blending to fabricate the testing specimens.

To our knowledge, there are currently no contributions on the processing of PLA/PCL/GNP composites using the 3D printing technique and especially leading to good electrical conductivity. Therefore, this work reports for the first time the fabrication of electrically conductive PLA/PCL/GNP 3D-printed materials that are able to connect an electrical circuit. Using the melt blending technique, PLA and PCL were mixed with GNP and were later extruded in the form of filaments. Eventually, the filaments were processed by 3D printing using the FFF technique. Later, the synthesized 3D-printed materials underwent contact angle measurement and rheological, thermal, mechanical, and electrical resistance tests, in addition to the microstructural analysis. For comparison, samples manufactured by injection moulding were prepared to assess the potential effects of the printing process on the microstructural and electrical properties of the composites.

## 2. Materials and Methods

### 2.1. Materials

PLA used in this work is a commercial grade Ingeo^TM^ Biopolymer 2003D (MFR = 6 g/10 min (210 °C/2.16 kg) supplied by NatureWorks (Minnetonka, MN, USA). Its main properties are a density of 1.2 g.cm^−3^, a glass transition temperature of 60 °C, and a melting temperature of 150 °C.

PCL is a commercial grade Capa^TM^ 6800 (MFR = 2.01–4.03 g/10 min (160 °C/5 kg) supplied by Perstorp UK Ltd. (Warrington, UK) in pellet form. PCL main thermal transitions are a glass transition temperature (Tg) of −60 °C and a melting temperature (Tm) in the 58–60 °C range.

Graphene nanoplatelets (GNP), trade name xGnP^®^, Grade M5, were supplied by XG Sciences Inc., Lansing, MI, USA. For this grade, the manufacturer reports an average lateral dimension of 5 µm, a thickness in the range of 6–8 nm, and a surface area of 120–150 m^2^.g^−1^. From the technical data sheet, some additional information regarding this material’s characteristics are available, such as: oxygen content less than 1% and residual acid content less than 0.5 wt.%. It features naturally occurring functional groups such as ether, carboxyl, or hydroxyl that can react with the atmospheric humidity to form acids or other compounds. Its density is 2.2 g.cm^−3^, thermal conductivity is 3000 W.m^−1^.K^−1^, thermal expansion of 4–6 × 10^−6^ K^−1^, tensile modulus of 10^3^ MPa, tensile strength of 5 MPa, and electrical conductivity of 10^7^ S.m^−1^. 

Diiodomethane (99% purity) used in this study was purchased from Alfa Aesar and used as received.

### 2.2. Samples’ Preparation

#### 2.2.1. Compounding

To prevent hydrolytic degradation, which is an undesirable reaction, PCL, PLA and GNP were dried at 40, 60 and 80 °C, respectively, overnight under vacuum before the melt mixing process. Melt blending using a co-rotating twin screw extruder Process 11 (Thermo Scientific, MA, USA) was carried out to produce the polymer blend and the composites. Before the processing step, PLA and PCL pellets were mixed manually and then placed in the extruder’s pellet feeder, whereas the GNP were placed in the extruder’s filler feeder. Next, during the extrusion process, all the materials were fed at the same time to produce a compounded wire that was immediately ground to form composite pellets. A PLA-PCL binary blend (80 wt.% of PLA and 20 wt.% of PCL) and PLA-PCL-GNP composites containing 10 wt.%, 15 wt.%, 20 wt.%, and 25 wt.% of M5 nanofillers were prepared. It is important to note that the weight percentages of PLA and PCL were fixed at 80 wt.% and 20 wt.%, respectively, with respect to the total polymer weight percentage in the polymer composites. The temperature profile of the twin-screw extruder was chosen as the following: 200 °C, 200 °C, 200 °C, 200 °C, 200 °C, 180 °C, 120 °C, and 70 °C set on the screw die zone and zones 8, 7, 6, 5, 4, 3, and 2 respectively. Note that zone 2 is the closest to the polymer feeder and zone 8 is the farthest. The rotor screw speed was 350 rpm, the total flow rate of the materials was 0.6 kg.h^−1^, and 300 g of materials were produced per composition.

#### 2.2.2. Injection Moulding

Following the twin-screw extrusion and after drying the composite pellets at 50 °C using a vacuum oven for at least one night, injection moulding was performed to manufacture samples for the electrical resistance characterizations. A mini-injection-moulding machine IM15 (Zamak Mercator, Skawina, Poland) was used and the parameters were: cylinder temperature of 180 °C, mould temperature of 60 °C, pressure of five bars, and cycle duration of 3 min. The samples produced for the electrical resistance tests were 1BA dumb-bell-shaped samples (according to the standard EN ISO 527-2), whose middle part was cut to produce bar-shaped samples of 30 mm length, 5 mm width, and 2 mm thickness.

#### 2.2.3. Filament Extrusion

Compounded materials were also used to feed a mini single-screw extruder NEXT 1.0 (3devo, Utrecht, Netherlands) in order to obtain calibrated filaments. The processing temperature profile gradually increased from T1 = 165 °C to T2 = 190 °C, T3 = 200 °C and T4 = 195 °C. Note that T1 is the temperature of the zone that is closest to the feeder and the screw rotation speed was fixed at 5 rpm. The final diameter of the extruded filaments was approximately 2.85 mm.

#### 2.2.4. Fused Filament Fabrication

The 3D-printed specimens were manufactured by an A4v3 FFF printer (3ntr, 28047 Oleggio NO, Italy), fed with the filaments extruded as described in the previous part. The 3D-printed samples were built in a horizontal configuration. All the specimens were 3D-printed using the following printing parameters: nozzle diameter of 0.8 mm, layer height of 0.2 mm, infill percentage of 100%, raster angle alternating between +45° and −45°, and infill speed of 40 mm.s^−1^. The 3D-printed parts were built at a nozzle temperature of 180 °C and at a bed temperature of 60 °C.

**Figure 1:**
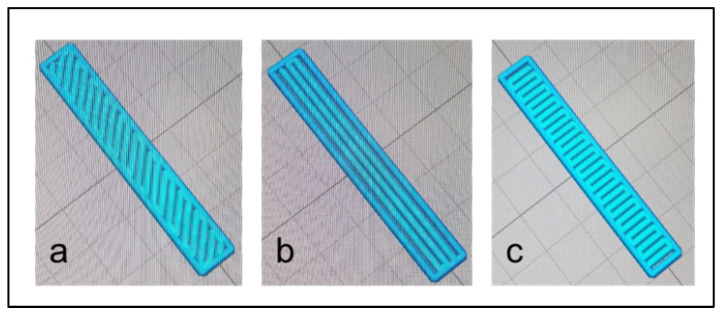
The different 3D printing patterns according to the three different raster angles: (**a**) +45°/−45°, (**b**) 0°, and (**c**) 90°.

The 3D-printed geometries of the samples were 25 mm disks for the rheological and contact angle measurements, ISO 527-1BA samples for the mechanical tensile tests, and bar shaped samples for the electrical resistance measurements (30 mm long, 5 mm wide, and 2 mm thick). The bar-shaped 3D-printed sample dimensions were chosen to be the same as those of the injection-moulded samples to perform a comparative study. Moreover, for the purpose of studying the influence of the printing pattern on the electrical conductivity results, the raster angle was varied and three values were chosen: +45°/−45° (Figure 1a), 0° (Figure 1b), and 90° (Figure 1c).

### 2.3. Characterization Techniques

#### 2.3.1. Contact Angle Measurements

The contact angle (θ) is the angle between the solid surface and the tangent drawn on the surface of a drop, passing through a triple intersection point between the atmosphere, liquid, and the solid. The θ values of probe liquids on PLA, PCL, and M5 surfaces took place at room temperature using a Krüss DSA30 goniometer. Measurements were performed using the sessile drop technique and a video-camera mounted on a microscope was used to record the drop images. Purified water (high-polarity) and diiodomethane (completely dispersive) with known surface tension polar and dispersive components were used as liquid probes. A drop of the probe liquid (3 μL for water and 1.5 μL for diiodomethane) on the sample’s surface was ejected. When the liquid drop settled, ten θ values were taken consecutively and their average value was calculated. 

The solid surface energy of a polymer is an important parameter that affects surface-related processes, such as wettability. Performing θ measurements using probe liquids with well-known surface tension properties is the most practical way to determine the solid surface energy. The solid surface energy can be obtained from the contact angle using Young’s equation (Equation (1)) [24]:

(1) $${ɣ}_{s}={ɣ}_{l}\cos\theta +{ɣ}_{\mathrm{sl}},$$

where ${ɣ}_{s}$ is the solid surface energy, ${ɣ}_{l}$ is the liquid surface tension, θ is the probe liquid contact angle, and ${ɣ}_{\mathrm{sl}}$ is the solid-liquid interfacial energy. The values θ and ${ɣ}_{l}$ are the only measurable quantities in Young’s equation. To determine ${ɣ}_{s}$, its polar and dispersive components should be calculated first using Owens–Wendt model using contact angles with at least two probe liquids (Equation (2)) [25]:

(2) $$\frac{{ɣ}_{l}\;\left(1+\cos\theta \right)}{2\sqrt{{ɣ}_{l}^{d}}}=\sqrt{{ɣ}_{s}^{p}}\times \frac{\sqrt{{ɣ}_{l}^{p}}}{\sqrt{{ɣ}_{l}^{d}}}+\sqrt{{ɣ}_{s}^{d}},$$

where ${ɣ}_{l}^{p}$ is the liquid polar surface tension, ${ɣ}_{l}^{d}$ is the liquid dispersive surface tension,$\;{ɣ}_{s}^{p}$ is the solid polar surface energy, and$\;{ɣ}_{s}^{d}$ is the solid dispersive surface energy. The total surface energy or tension is therefore the sum of the polar and dispersive surface energies or tensions.

The PLA and PCL samples for these tests were 3D-printed 25 mm diameter disks, whose smooth side was tested, and the M5 samples was pressed into compact 25 mm disks. Six sessile drop tests were performed for each liquid and each sample and two samples were tested per material. It is important to note that for the M5 GNP samples, the contact angles recorded in the first three seconds were taken into consideration as a first approximation of surface energy determination after each drop ejection, due to the absorptive capacity of the sample that is in a powdered form. 

The liquid surface tensions and their polar and dispersive components of the test liquids are presented in Table 1 [26]. The solid-surface energies of PLA, PCL, and M5 GNP can be used to calculate the interfacial energies between each two materials and, eventually, to estimate the wetting coefficient that can be used to predict the selective localization of the fillers in the polymer blend composites. In this context, the formulas that can be used for these calculations are listed below [27]:

(3) $${ɣ}_{\mathrm{ij}}={ɣ}_{i}+{ɣ}_{j}-\frac{4{ɣ}_{i}^{d}{ɣ}_{j}^{d}}{{ɣ}_{i}^{d}+{ɣ}_{j}^{d}}-\frac{4{ɣ}_{i}^{p}{ɣ}_{j}^{p}}{{ɣ}_{i}^{p}+{ɣ}_{j}^{p}},$$

(4) $${ɣ}_{\mathrm{ij}}={ɣ}_{i}+{ɣ}_{j}-2\sqrt{{ɣ}_{i}^{d}{ɣ}_{j}^{d}}-2\sqrt{{ɣ}_{i}^{p}{ɣ}_{j}^{p}},$$

Equation (3) is Wu’s harmonic mean equation and is used to calculate the interfacial tension ${ɣ}_{\mathrm{ij}}$ between PLA and PCL using the polymers surface energies and their dispersive and polar components. For the calculation of the interfacial tension between the filler and the polymers, Wu’s geometric mean equation (Equation (4)) can be used, where ${ɣ}_{i}^{d}$ and ${ɣ}_{i}^{p}$ are the dispersive and polar contributions to the total surface tension ${ɣ}_{i}$, respectively. The interfacial tensions between PLA and PCL (${ɣ}_{\mathrm{PLA}/\mathrm{PCL}}$) and between M5 and the polymers (${ɣ}_{M5/\mathrm{PLA}}$ and ${ɣ}_{M5/\mathrm{PCL}}$) are therefore useful for the determination of the wetting coefficient ($\omega_{\alpha })$ [27]:

(5) $$\omega_{\alpha }=\cos\theta =\frac{{ɣ}_{M5/\mathrm{PCL}}-{ɣ}_{M5/\mathrm{PLA}}}{{ɣ}_{\mathrm{PLA}/\mathrm{PCL}}},$$

If the wetting coefficient ($\omega_{\alpha }$) is greater than one, then M5 GNP should be in PLA. If it is smaller than −1, then M5 GNP should be localized in PCL. If ($\omega_{\alpha }$) is between 1 and −1, then M5 should be located at the interface of the two polymers. 

#### 2.3.2. Scanning Electron Microscopy (SEM)

The morphologies of the neat GNP and the injection-moulded and 3D-printed PLA/PCL/10 wt.% M5 and PLA/PCL/15 wt.% M5 samples were analysed at different levels of magnification by a scanning electron microscope (FEI Quanta 200 SEM) (Fisher Thermo Scientific) at an acceleration voltage of 10 kV. The analysis was preceded by cryofractures of cross-sections using nitrogen. To assess the affinity of the GNP to the polymer phases, the cryofractured injection-moulded and 3D-printed PLA/PCL/10 wt.% M5 and PLA/PCL/15 wt.% M5 samples were immersed in toluene at room temperature for 3 h in order to dissolve the PCL phase, followed by washing to remove the dissolved fraction. Samples were then placed under the hood overnight for complete solvent evaporation prior to the SEM analysis. The observation of the bar-shaped samples, whether dissolved in toluene or not, took place along their transversal section.

#### 2.3.3. Atomic Force Microscopy (AFM)

The morphology and microstructure of the injection-moulded and 3D-printed PLA/PCL/10 wt.% M5 composites were investigated by the atomic force microscopy (AFM) technique (MFP-3D infinity, Asylum Research AFM, Oxford instruments, Abingdon, UK). The samples’ surfaces were cryogenically sectioned by diamond knifes using an ultra-microtome (Leica EM UC7, Nanterre, France) to obtain flat and smooth surfaces. AFM characterisations were carried out along the transversal section of the bar-shaped samples. In addition, AFM images were obtained in a tapping mode under ambient conditions using AC160TSR3 tip at a scan rate of 1 Hz. Images were created at different sizes, from 3 × 3 to 20 × 20 µm^2^. Both the topographic (height) and phase images were recorded and analysed. 

#### 2.3.4. Rheological Measurements

The rheological measurements were carried out in oscillatory shear mode using parallel plate geometry (25 mm diameter) rheometer (Anton Paar, Graz, Austria) at 190 °C under nitrogen atmosphere to prevent sample degradation. Frequency sweeps between 0.01 and 600 rad.s^−1^ were carried out at low strains (0.006–10%), which were shown to be within the linear viscoelastic range of these materials. The upper limits of the viscoelastic range were determined by strain sweep tests at 200 rad.s^−1^. Before testing, the PLA/PCL blends and the composites were vacuum-dried overnight at 50 °C and the PLA and PCL pure samples were also vacuum-dried at 60 °C and 40 °C respectively. The tests were repeated two times for each composition to assess reproducibility.

#### 2.3.5. Thermogravimetric Analysis (TGA)

Thermogravimetric Analysis (TGA) was carried out on 10 mg samples using a TGA Perkin Elmer apparatus at 10 °C.min^−1^ from 30 °C to 900 °C with nitrogen flow (40 mL.min^−1^). This step was preceded by an isothermal step for 30 min at room temperature. The weight loss was recorded as a function of temperature. The experimental percentage of char yield at 600 °C, the thermal onset degradation temperature at 5% of mass loss (T_onset_), the maximum degradation temperature T_max_ (i.e., temperature of the mass loss rate peak), and the total mass loss over the entire temperature range were determined.

#### 2.3.6. Differential Scanning Calorimetry (DSC)

Thermal analysis was performed by means of a Perkin-Elmer Diamond differential scanning calorimeter (DSC). The samples were sealed in aluminium pans (10 mg) and then heated from room temperature to 200 °C, held for 5 min to eliminate previous thermal histories, cooled down to room temperature, and finally re-heated to 200 °C. A scanning rate of 10 °C.min^−1^ was used in both heating steps and the cooling rate was chosen to be 5 °C.min^−1^. The glass transition temperature, T_g_, was measured at the mid-point of the heat capacity inflexion point. The crystallization temperature, T_c_, and melting temperature, T_m_, were determined from the peak values of the respective exotherms and endotherms. The degree of crystallinity of PLA in the samples (% Xc) was determined by means of Equation (6):

(6) $$\%\;\mathrm{Xc}=100\times \frac{\Delta H_{m}-\Delta H_{c}}{\Delta H_{m}^{0}\cdot \;W},$$

where $\Delta H_{m}^{0\;}$ is the enthalpy value of a pure crystalline PLA corresponding to 93.7 kJ.mol^−1^ [28], ΔH_m_ is the enthalpy of PLA related to the fusion process, ΔH_c_ is the enthalpy of PLA related to the crystallization process, and W is the weight fraction of PLA in the blend and the composites.

#### 2.3.7. Tensile Tests

Tensile tests were carried out using a static materials testing machine ZwickRoell BZ010/TH2S (ZwickRoell, Ulm, Germany) equipped with a 2.5 kN load cell on dumbbell-shaped specimens (geometry type 1BA, ISO 527). For the assessment of Young’s modulus, the strain in the linear range was determined at a cross-head speed of 1 mm/min^−1^ by means of a clip-on extensometer. The yield stress and the elongation at break were determined at a cross-head speed of 10 mm.min^−1^. Five 3D-printed tensile specimens were tested for each formulation: PLA, PLA/PCL, and the composites.

#### 2.3.8. Electrical Resistance Measurement

**Figure 2:**
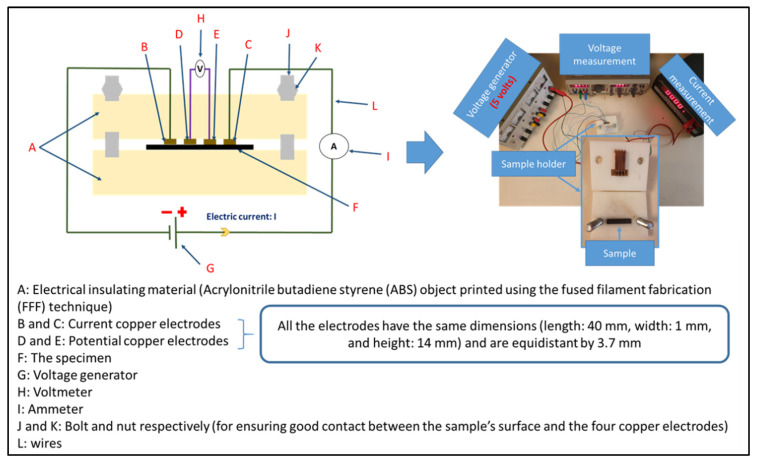
The setup of the electrical resistance measurement using the four probes method.

For moderately conductive materials (whose volume resistivity ranges between 1 and 10^7^ Ω.cm), volume resistivity was determined using four-probe contact configuration. The resistance measurement was performed on six bar-shaped samples for each formulation. Each specimen was tested at a voltage of 5 V by using a direct current (DC) power supply (Topward, TPS-4000). The current flow on the samples was measured between two external electrodes using a multimeter (MX 579 Metrix, ITT instruments, New York, NY, USA) and the voltage was measured between the internal electrodes using a digital multimeter (HM 8011, HAMEG Instruments, Mainhausen, Germany). One surface of each sample was in contact with the four identical copper electrodes, whose dimensions were: length of 40 mm, width of 14 mm, and thickness of 1 mm. The electrodes were equidistant from each other with 3.7 mm. The resistance measurement setup is shown in Figure 2. The electrical volume resistivity (ρ) was calculated according to Equation (7):

(7) $$\rho =\frac{R\times S\;}{L},$$

where *R* is the electrical resistance (Ω), *S* is the cross-sectional area (m^2^), which is equal to the width of the sample multiplied by its thickness, and *L* corresponds to the distance between the internal electrodes (3.7 mm).

## 3. Results and Discussion

### 3.1. Contact Angle Measurement

Based on the contact angle measurements performed in this study, the PLA and PCL possess solid surface energies equal to 39.4 ± 0.6 mJ.m^−2^ and 35.5 ± 0.2 mJ.m^−2^, respectively. These results are shown in Table 2 and compared to results in previous research for the same materials. The values found in previous research are similar to ours for the three materials (polymers and graphene), although the experimentation conditions and/or the models used for calculating the surface energies are not the same. Furthermore, the results for M5 GNP are also presented; the solid surface energy value obtained by our measurements is 58.9 ± 0.1 mJ.m^−2^. 

The calculations of the interfacial surface energies between PLA and PCL, PLA and M5, and PCL and M5 based on Equations (3) and (4) in the characterizations section produced the following results, respectively: ${ɣ}_{\mathrm{PLA}/\mathrm{PCL}}\;$= 0.424 mJ.m^−2^,$\;{ɣ}_{M5/\mathrm{PLA}}\;$= 8.835 mJ.m^−2^, and ${ɣ}_{M5/\mathrm{PCL}}\;$= 11.468 mJ.m^−2^. Consequently, the wetting coefficient based on these results and calculated according to Equation (5) in the characterizations section is 6.2 (greater than one), indicating that in principle, M5 GNP should be localized in PLA. However, it is important to take into consideration that the thermodynamic factor is not the only factor that affects the localization of the fillers in the polymer blends. Some other factors, such as kinetic factors, exert an influence on the filler localization phenomenon. For instance, the viscosity ratio between the polymers, the temperature, the time of mixing, the sequence of mixing of the components, and the shear rate are examples of these factors [29].

### 3.2. Microscopy-SEM and AFM

**Figure 3:**
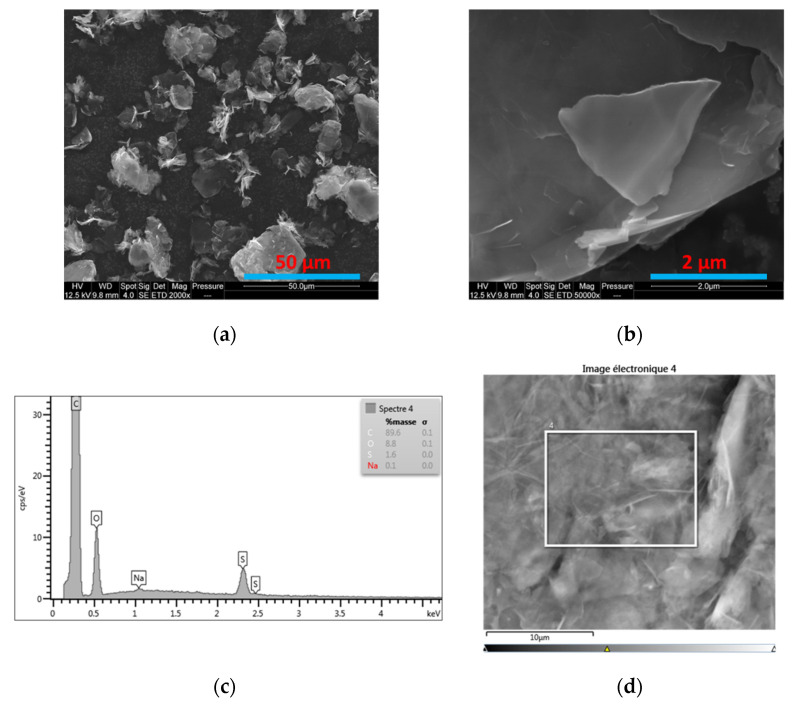
(**a**,**b**) SEM images of M5 GNP at different magnifications; (**c**) EDX graph; (**d**) the part of the surface that is surrounded by a rectangle and whose surface elements are shown in the EDX graph (**c**).

Figure 3 corresponds to the scanning electron microscopy investigations carried out on the M5 GNP. In particular, information about the morphology of M5 GNP is given by Figure 3a,b, which clearly show the platelet form of graphene. Figure 3c represents the energy-dispersive X-ray spectroscopy (EDX) analysis of the M5 GNP surface to figure out the elements composing the zone indicated by the square shown in Figure 3d. These elements are mainly carbon (89.6%) and oxygen (8.8%). Other elements were also identified, such as sulfur (1.6%) and sodium (0.1%). The M5 oxygen content can be attributed to the presence of carbonyl and ether/alcohol groups [36].

**Figure 4:**
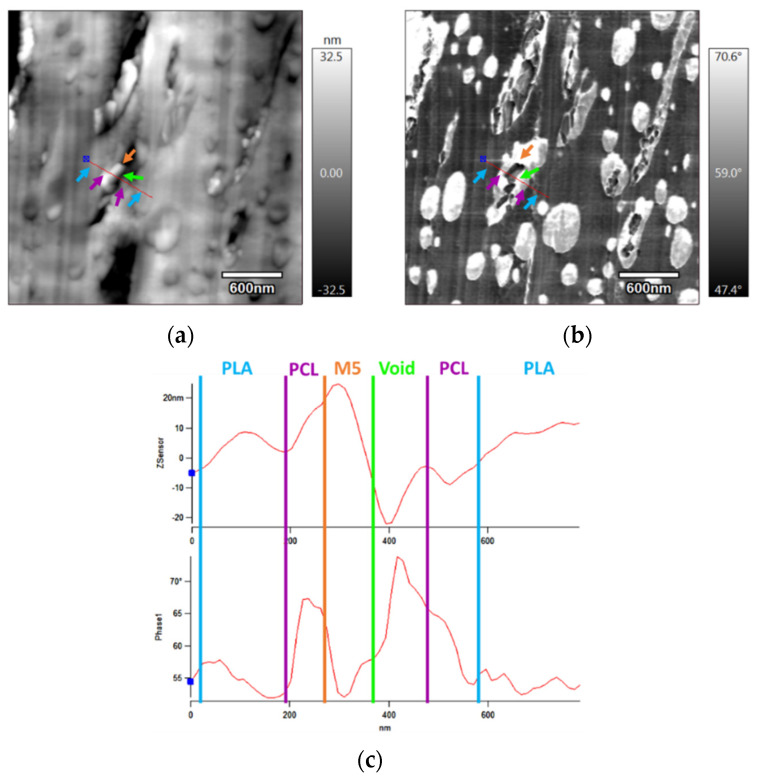
**(a**) AFM topography; (**b**) AFM phase images of the PLA/PCL/10 wt.% M5 injection-moulded sample; (**c**) topography and phase profiles on a drawn red line that is traced in (**a**,**b**) (the fleshes in the images point towards the different components of the composite and their colours match the designation of the peaks in the profiles).

Atomic force microscopy (AFM) was performed on both injection-moulded and 3D-printed samples in order to investigate the influence of the process on the morphology of these composites. Figure 4a,b exhibit the topography and the phase of the injection-moulded sample, respectively. The topography (Figure 4a) showed that the roughness of the surface was quite low (<100 nm), with some rough (white) zones highlighting the presence of GNP. Phase contrasts (Figure 4b) provided additional information: white nodules related to PCL (with the highest phase) are clearly visible in a grey matrix (denoting the PLA). Graphene is also visible (in dark grey). To identify more precisely the elements of the composite, a profile was drawn on the images. Figure 4c shows these profiles, which correspond to the red line that is displayed in Figure 4a,b, starting from the blue point (that is present in the images and the profiles). Along the drawn line, the three composite components are indicated by arrows whose colours match the colour of the designations in the profiles. The components were named by considering that in the phase profile, the greatest peak corresponds to the softest material, the PCL, and the lowest peak refers to the stiffest materials, the graphene nanoplatelets. However, this is the inverse for the topography profile. The combination of the information from these figures confirms that the small white nodules are PCL, the grey matrix is the PLA that is present in the greatest proportion, and the layered structured material, in dark grey, corresponds to the lowest phase, the graphene nanoplatelets.

**Figure 5:**
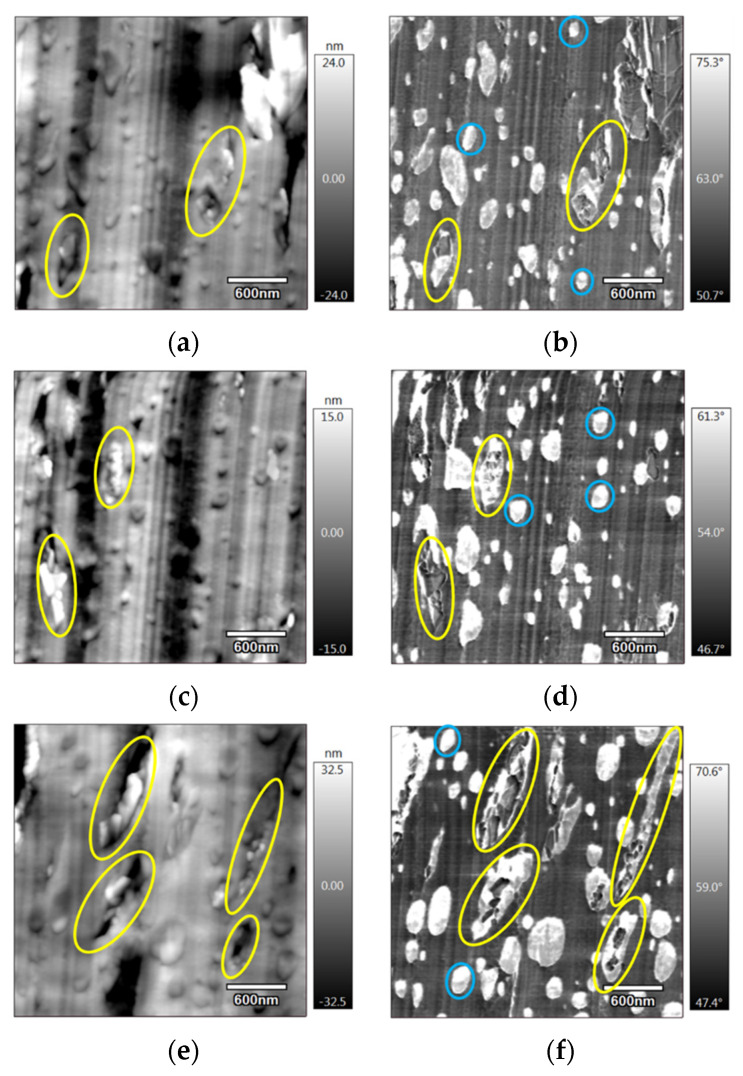
(**a**,**c**,**e**) AFM topography; (**b**,**d**,**f**) the corresponding phase images of (**a**,**c**,**e**), respectively, of the injection-moulded PLA/PCL/10 wt.% M5 (the yellow circles surround the PCL particles containing GNPs and the blue circles surround the pure PCL nodules).

Figure 5 also corresponds to an injection-moulded PLA/PCL/10 wt.% M5 composite. In both their topography (Figure 5a,c,e) and their corresponding phase images (Figure 5b,d,f), the yellow circles surround some graphene particles that seem to be localized in the PCL nodules. This selective localization of the graphene in the PCL phase rather than the PLA phase was performed in a work carried out by De Aguiar et al. [37]. In their work, they stated that the simultaneous melt blending of two polymers, such as PLA and PCL, whose weight percentages were 80 wt.% and 20 wt.% respectively, characterized by melting temperatures of 155 °C and 60 °C, respectively, drives the nanoparticles to the PCL phase. The PLA’s viscosity is greater than that of the PCL for all the analyzed shear rates and this difference determines the preference of the nanoparticles for the lower-viscosity polymer phase. Furthermore, due to the slight differences in terms of the interfacial tensions between PLA/GNP (0.82 mJ.m^−2^) and PCL/GNP (0.3 mJ.m^−2^), the viscosity effects dominate. In the present study, and based on the contact angle measurements and the wettability calculations, the M5 GNP was expected to be localized in the PLA phase. However, Figure 5 shows that this is not the case, and that M5 GNP prefers PCL. This can be explained by the dominance of the kinetic factors, such as the viscosity, over the thermodynamic factors. Moreover, it was highlighted by Kelnar et al. [38] that the addition of GNP to the PLA80/PCL20 blend showed the significant presence of GNP in the PCL phase and a limited presence at the interface. The authors explained that this phenomenon was due the initial dispersion of the nanoparticles in the first melting polymer phase, PCL, during the one-step melt mixing process. Furthermore, due to the similarity between the interfacial energies of PLA, PCL, and GNP, there is no sufficient driving force that can cause the transfer of graphene to PLA. It is also important to note the circular form of the PCL nodules, indicated by blue circles in Figure 5b,d,f. When the M5 particles are situated in these nodules, their size becomes bigger and their form becomes more elongated (yellow circles in Figure 5b,d,f). This can be attributed to the aggregation of the nanoparticles and their alignment inside the PCL nodules and can also be the result of the alteration of the polymer viscosity ratio by the nanoparticles present in one of the polymer phases [37].

**Figure 6:**
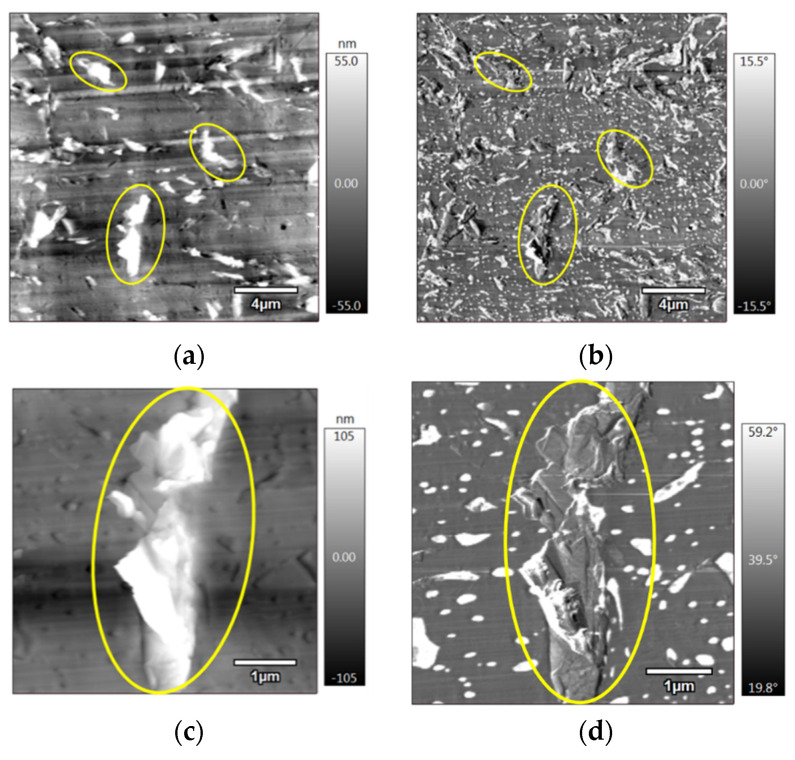
(**a**) AFM topography; (**b**) AFM phase, (**c**) zoomed image of (**a**); (**d**) zoomed image of (**b**), of 3D-printed PLA/PCL/10 wt.% M5 (the yellow circles surround few GNPs).

Figure 6 shows the AFM images of the 3D-printed PLA/PCL/10 wt.% M5 composite, where Figure 6a and its zoomed image, Figure 6c, correspond to the topography images and Figure 6b and its zoomed image, Figure 6d, are the relevant phase images. The yellow circles surround some graphene particles. The absence or low presence of the graphene particles inside the PCL nodules is noticeable if compared to previous observations of the injection-moulded samples. This might be due to the formation of more graphene aggregates in the 3D-printed samples, which hindered their positioning in the small PCL nodules; instead they were all distributed in the PLA matrix. Lower mixing and laminar flow during the fabrication of filaments prior to printing could cause aggregations owing to the action of van der Waals interactions between the graphene particles. The zoomed images, Figure 6c,d, show an example of these aggregates surrounded by a yellow circle.

**Figure 7:**
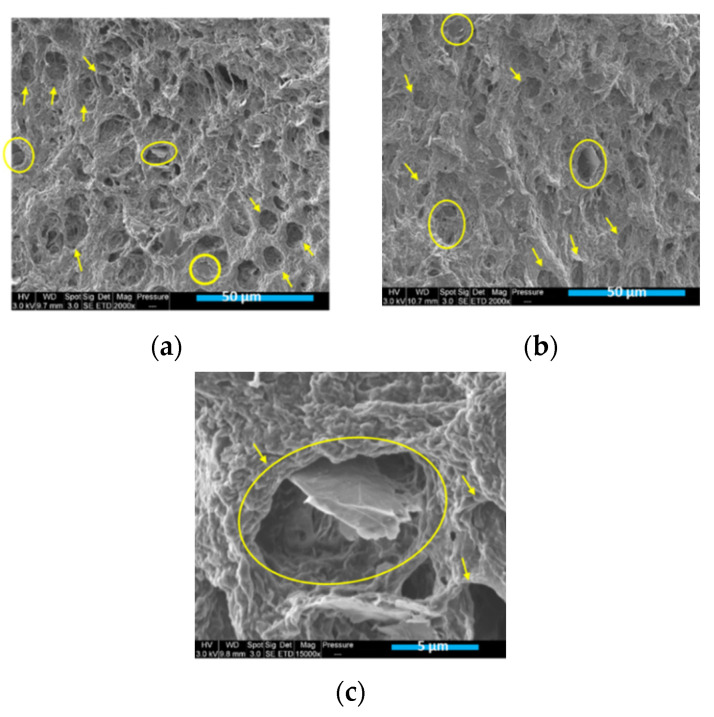
(**a**,**c**) SEM images of toluene-etched, injection-moulded PLA/PCL/10 wt.% M5 composite; (**b**) SEM images of toluene-etched, 3D-printed PLA/PCL/10 wt.% M5 composite (The yellow circles surround the GNPs positioned in the pores where the PCL nodules were expected to be located and the yellow fleshes are pointed towards these pores).

The SEM images of the PLA/PCL/10 wt.% M5 injection-moulded and 3D-printed samples are exhibited in Figure 7. Prior to the SEM analysis, the surfaces of these samples were etched using toluene, which is able to dissolve only the PCL phase at room temperature, while it exerts no influence on the PLA phase. These images are in accordance with the AFM observations, in all of which the yellow arrows are pointed towards the pores where PCLs were supposed to be present, emphasizing the nodular structure of this polymer in the composites. In addition, the yellow circles surround some of the graphene flakes that are present in the PCL pores, thus confirming what has been indicated by the AFM technique regarding the preference of this nanofiller to the PCL phase.

**Figure 8:**
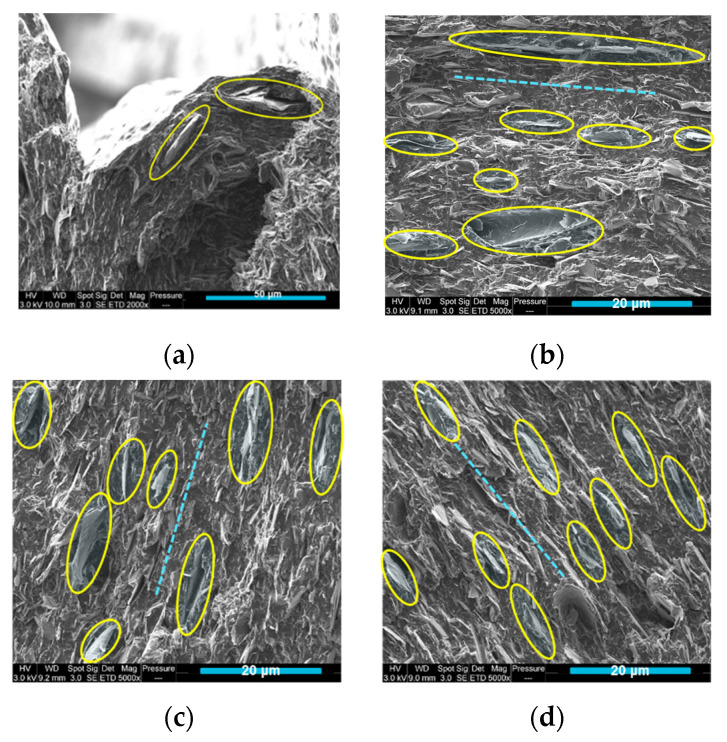
(**a**) SEM image of 3D-printed PLA/PCL/15 wt% M5; (**b**–**d**) SEM images of injection-moulded PLA/PCL/15 wt.% M5 composite (The yellow ellipses are surround some graphene flakes and the dotted lines indicate the similar orientation of the graphene platelets in the injection moulded samples).

In Figure 8, several SEM images of the injection-moulded (b, c, d) and 3D-printed PLA/PCL/15 wt.% M5 (a) composites are shown. The yellow ellipses in Figure 8a surround some graphene platelets whose orientation is driven by the printing pattern. The extrusion-induced orientation phenomenon of GNPs is due to the large shear rate between the molten composite and the inner wall of the nozzle [39]. In Figure 8b–d, the doted blue lines show that most of the graphene nanoplatelets (surrounded by yellow circles) featured similar orientations in these parts of the injected samples, as determined by the flow direction of the molten material during the injection process. Wu and Drzal compounded GNP and polyetherimide (PEId) by melt-extrusion followed by injection molding. They figured out that the GNP have unidirectional orientation in the injection-moulded specimens [40].

**Figure 9:**
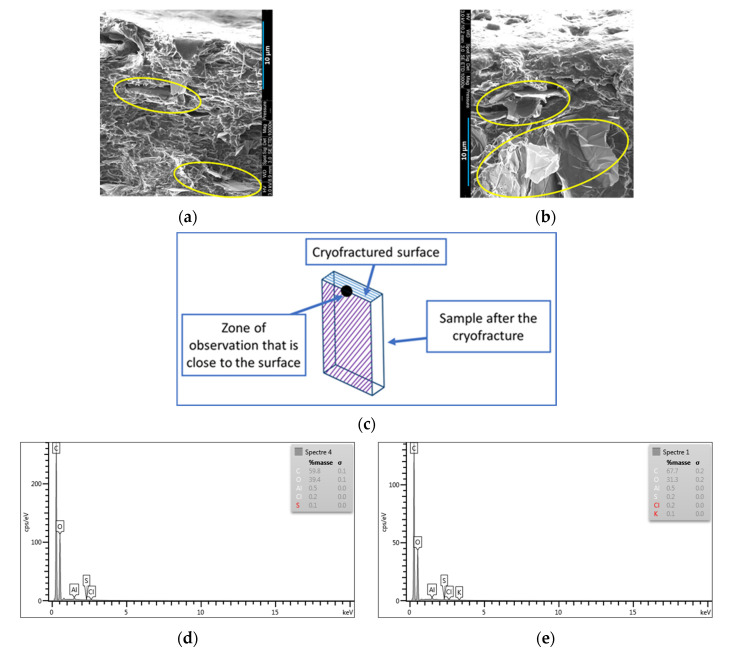
(**a**) SEM image of the injection-moulded PLA/PCL/15 wt.% M5 composite and its EDX (**d**); (**b**) SEM image of 3D printed PLA/PCL/15 wt.% M5 composite and its EDX (**e**); (**c**) the zone of observation where (**a**,**b**) were determined and indicated by the small black circle (the yellow circles surround some graphene flakes).

Figure 9a,b correspond to the SEM pictures of the injection-moulded and 3D-printed PLA/PCL/15 wt.% M5 specimens, respectively. These images are relevant to the parts close to the surface (small black circle in Figure 9c). What is clear is the lower presence of graphene close to the surface of the injection-moulded sample compared to the 3D-printed sample (graphene particles are surrounded by yellow circles); this is supported by the EDX spectra (Figure 9d,e). The EDX of the 3D-printed sample shows greater carbon content (66.2%) than the EDX of the injection-moulded sample (60.2%); this confirms the higher presence of GNP on the surface of the 3D-printed sample.

**Figure 10:**
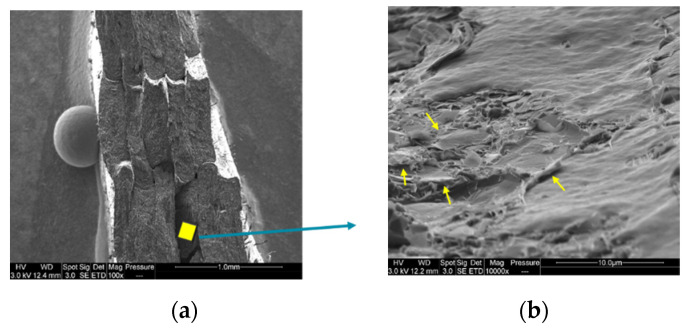

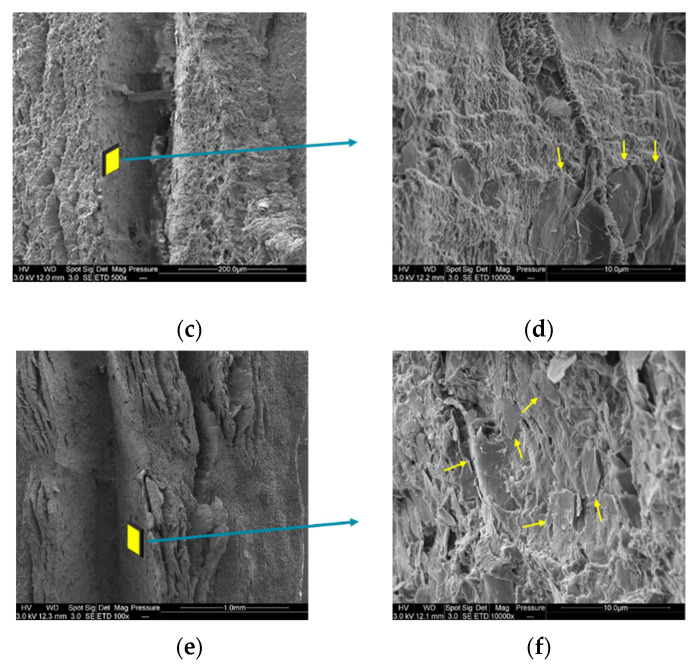
(**a**,**c**,**e**) SEM images at the interface of two 3D printed filaments of PLA/PCL/15 wt.% M5 composites; (**b**,**d**,**f**) the zoomed images of (**a**,**c**,**e**), respectively (the yellow arrows are pointed towards some graphene nanoplatelets).

Figure 10 shows the interface of two 3D-printed filaments of non-etched (Figure 10a,b) and etched (Figure 10c–f) surface samples. The yellow rectangles in Figure 10a, Figure 10c, and Figure 10e are zoomed in Figure 10b, Figure 10d, and Figure 10f, respectively. In the zoomed images, the yellow arrows are pointed towards stacks of graphene flakes present at the interface of two printed filaments in the final 3D-printed part. During the printing process, the material is deposited in the form of thin filaments and, at this point, the graphene flakes gather at the interface.

### 3.3. Rheology Results of 3D-Printed Samples

The visco-elasticity measurements of the PLA/PCL/M5 nanocomposites were performed to study the influence of GNP loading on the performance of the composites as well as to study the morphology and the formation of the internal graphene network within the manufactured composite systems. The state of dispersion and interactions between polymer/nanoparticles are closely related to their rheological properties [41]; therefore, the distribution of the nanoparticles in the polymer matrix was examined using this measurement technique.

**Figure 11:**
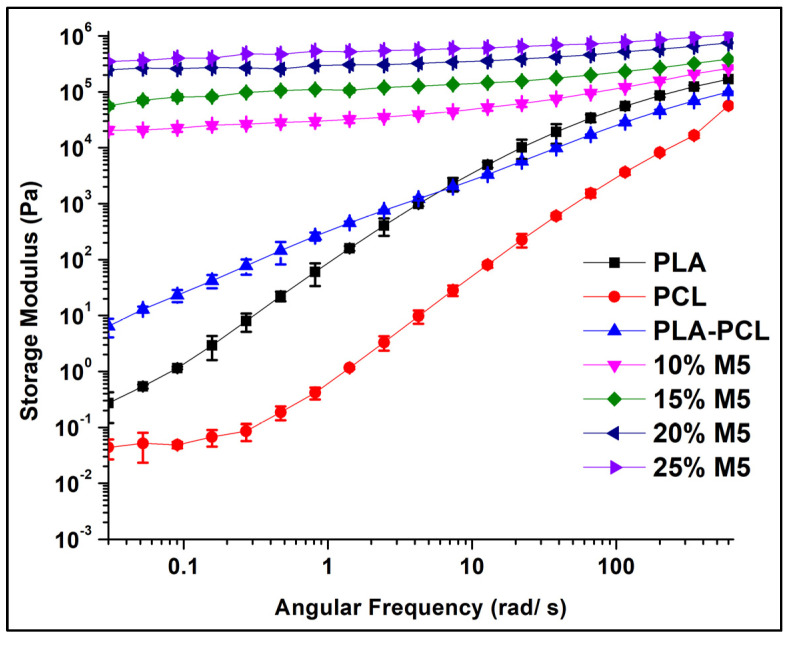
The storage modulus curves of PLA, PCL, and PLA/PCLs blends and their composites.

**Figure 12:**
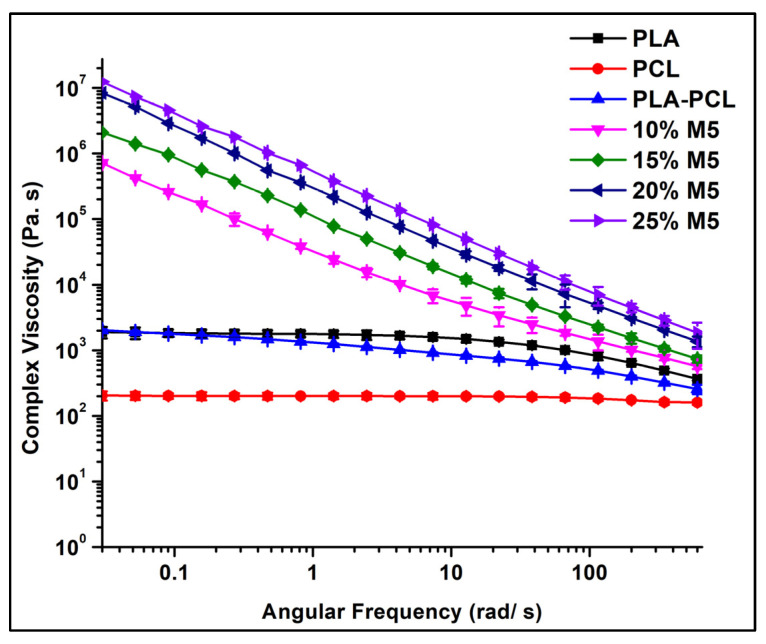
The complex viscosity curves of pure PLA and PCL polymers in addition to their blend and composites.

The storage modulus (G’) and complex viscosity dependence on the angular frequency in the linear viscoelastic region at 190 °C are presented in Figure 11 and Figure 12, respectively. The pure polymers and the PLA/PCL blend exhibited terminal liquid-like behaviour or a classical viscoelastic response in the storage modulus curve, as shown in Figure 11. However, in the composites, the storage modulus exhibited independence from the angular frequency, which was illustrated by a plateau in the terminal or low-frequency region. This plateau indicates the formation of a non-terminal solid percolated network of the M5 GNP inside the polymer matrix. It is important to note that the storage modulus of the polymer composites increased with the increase in the percentage of graphene; this was due to the increased number of filler–filler interactions, as well as the reduction in the chain mobility of the polymers located near the graphene particles. Moreover, the length of the plateau extended to higher frequencies with the increase in the percentage of graphene; this also emphasizes the formation of filler networks and the entrapment of the polymeric chains in the filler network, decreasing the flow of the polymers at low frequencies [42].

As shown in Figure 12, the complex viscosity increased with the increase in the graphene percentage in the matrix. This increase was more pronounced at lower frequencies, and with gradual increases in the frequency, it was reduced due to the shear thinning behaviour [42]. The polymers and their blends exhibited Newtonian behaviour followed by shear-thinning behaviour, which is considered a common behaviour of thermoplastics. For the composites, this was not the case: there was no Newtonian behaviour and the increase in the viscosity at low frequencies probably indicates yield stress fluid behaviour.

Following the modulus and viscosity change in the low frequency region is important to determine the rheological percolation threshold of filled polymer composites. The complex viscosity increased sharply at 10 wt.% of M5, indicating that there was a sudden change in the material structure and that the material reached the point rheological percolation, at which point the filler impeded the motion of the polymer matrix and formed a nanoscale network structure. In addition, the storage modulus was independent of the angular frequency in the low-frequency region when the graphene percentage was 10 wt.%. This is an indication of the transition from liquid-like to solid-like viscoelastic behaviour, demonstrating that this graphene percentage is above the rheological percolation threshold [43].

### 3.4. Thermogravimetric Analysis (TGA) of 3D-Printed Samples 

**Figure 13:**
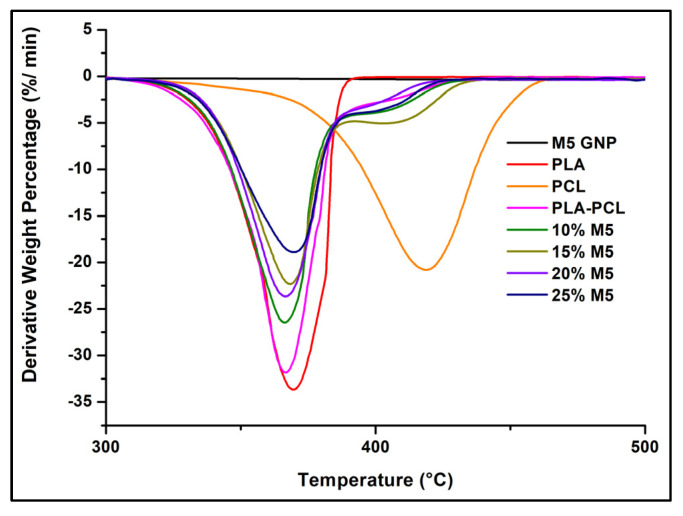
DTG curves (N2 atmosphere, 10 °C.min^−1^) of GNP, PLA, PCL, and PLA/PCL blends and their composites.

The thermal stability of the GNP, PLA, PCL, and PLA/PCL blends and their composites are shown in Figure 13 and Table 3. These results show that PCL features better thermal stability than PLA, as indicated by the higher onset and maximal degradation temperatures. The mixture of the PLA and PCL produced a blend with thermal properties closer to those of PLA than to those of PCL due to the greater weight percentage of PLA in the polymer blend. In the blend and composites, there were two steps of degradation, corresponding to the individual degradation of both non-miscible polymers. Furthermore, the comparison between the theoretical and experimental percentages of char yield at 600 °C for all the compositions shows that both were close. This indicates that there was no improvement in char formation and that there was a kind of dilution effect, which could be attributed to the formation of graphene aggregates in the polymer matrix. Regarding the thermal stability of the composites, normally, the GNP in these materials is prone to forming a thermally stable network around the polymer matrix, which could act as a protective layer. This layer is expected to lower the rate of heat transfer and the mass loss of the polymers. In addition, the GNPs’ interfacial interaction with the polymer matrix could increase the energy required for the polymers’ decomposition, leading to greater thermal stability of the composites compared to the polymers alone [44]. Furthermore, the radical scavenging function of GNP could inhibit the degradation process of the organic polymers [45]. For instance, with respect to the PLA/graphene and PCL/graphene systems, normally, the presence of graphene enhances the thermal stability of these polymers; this effect was observed in several studies [11,46,47,48,49,50,51,52,53]. However, in our case, the addition of M5 GNP to the PLA/PCL blend did not cause any improvement in thermal stability, regardless of the percentage of GNP. This may be attributed to the presence of aggregates of GNP and their poor interfacial adhesion to the polymer matrix.

### 3.5. Differential Scanning Calorimetry (DSC) of 3D-Printed Samples

**Figure 14:**
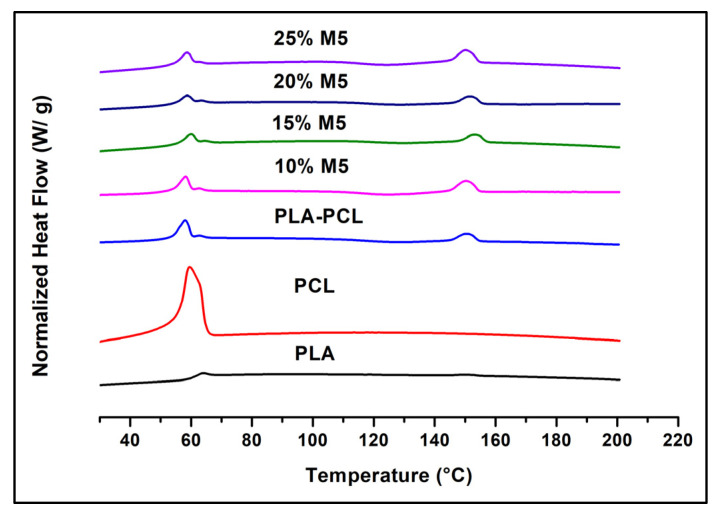
DSC curves of PLA, PCL, their blend, and their composites.

Figure 14 shows the DSC curves of PLA, PCL, their blend, and their composites, the results of which are summarized in Table 4. The results were taken from the second heating step. The presence of PCL with PLA improved its crystallinity due to the appearance of the PLA crystallization peak in the PLA/PCL blend and the increase in the area of its melting peak. A similar nucleating effect of PCL in PLA was obtained in some other works [4,54,55,56]; the reason behind this is the recrystallization of imperfect PLA crystals into more perfect α crystals in the presence of PCL [57]. Other authors interpreted this improvement in PLA crystallinity as being due to the increase in the number of PLA nuclei that are generated at low temperatures and induced by the presence of PCL [58]. The degree of crystallinity of PLA was more enhanced in the case of composites with the increase in the percentage of graphene compared to the pure blend. This indicates that the graphene exerted a slight nucleating effect on the PLA crystallites in the PLA/PCL matrix. The melting temperatures of PLA and PCL were unchanged in the case of the blends and composites compared to the pure polymers. Furthermore, the PLA crystallization temperature was the same for the blend and the composites.

### 3.6. Tensile Properties of 3D-Printed Samples

The mechanical properties of polymer composites are governed by various factors, such as the filler type and its structure and morphology, filler loading, filler quality, filler dispersion, filler orientation (random or aligned), filler–matrix adhesion, particle dimensions, voids, filler surface modification, and fabrication method [59]. Factors that can lead to improvements in the tensile properties of GNP composites include the homogeneous dispersion of the nanoparticles, their orientation in the polymer matrix, and the strong interfacial interaction between graphene and the matrix [60]. In addition, when inorganic fillers such as GNP are loaded into polymeric materials, they are capable of playing the role of the skeleton in the matrix. Therefore, the movement of the macromolecular chains is limited due to several physical cross-linking points between them and the polymer chains, thus improving the composite’s stiffness. The stiffness could also be improved by the heterogeneous nucleation performed by the filler, which causes an increase in the degree of crystallinity of the matrix or a change in its crystal-type structure [61]. 

Table 5 shows the tensile properties of the 3D-printed PLA, PLA/PCL blend, and PLA/PCL/M5 composites (raster angle: +45°/−45°). The Young’s modulus was enhanced in the composites compared to the pure blend. This increase shows that GNP exerts a stiffening effect on the PLA/PCL matrix.

When a polymer composite filled with inorganic thin sheet-shaped particles is under tensile load, the matrix generates relative deformation, whereas the particles do not deform [61]. The tensile strength could be decreased due to the geometry of the GNPs, which possess flaky structures and stack over each other to form agglomerations; it may also be due to the GNPs’ sharp edges, which initiate cracks within the matrix [59]. In our case, the maximal tensile strength decreased with the addition of graphene. Moreover, referring to the SEM images of the 3D-printed PLA/PCL/15 wt.% M5 composites (Figure 8a and Figure 9b), the limited adhesion between the graphene particles and the matrix is obvious. This can explain the decrease in the tensile strength of the composites. This decrease could also be due to the location of the GNP at the surface of the filaments (Figure 10), which can enhance the delamination of the 3D-printed adjacent layers. In a work focused on understanding the influence of the filler size on the properties of PLA/GNP nanocomposites, the tensile strength was increased for the composites containing 7 wt.% of filler, but beyond this value of weight percentage, the tensile strength was decreased due to the presence of aggregates [3].

Tensile elongation at break is an important parameter for characterizing the ductility or the tensile fracture toughness of materials. The elongation at break was enhanced only in the case of PLA/PCL blend compared to PLA alone. The increase in the graphene percentage led to a further decrease in this parameter. This result was also obtained by Gao et al. [3], who explained it by the fact that large GNP platelets exhibit a large interfacial surface area, which could make them more efficient at transferring stresses and thus at acting as stiffening fillers, but at the expense of the composite’s ductility. This can be due to the larger interfacial surface area (per plate), which causes higher local stress concentrations in the matrix and, thereby resulting in the embrittlement of the composite. Furthermore, composites’ ductility is affected by the state of dispersion of the GNP; more aggregations lead to reduced toughness.

### 3.7. Electrical Volume Resistivity

Conductive nanofillers provide many conductive pathways in insulating matrices, resulting in the increase in their electrical conductivity. For composites to be electrically conductive, the incorporated conductive fillers should make contact with one another and construct interconnected pathways for electrons to travel. Using fillers with large-sized sheets makes it possible for the pathways to be formed with low concentrations [62]. For instance, there are many types of contact between GNP particles in the polymer matrix, such as plane-to-plane, edge-to-edge, and edge-to-plane [63].

When the electrical resistance measurements were performed using the four-probes contact method on the injection-moulded and 3D-printed composites, there were no results for the injection-moulded samples. This was also determined by Wu et Drzal [40], who found much better electrical conductivity for compression-moulded GNP/polyetherimide (PEId) composites compared to injection-moulded composites (two times greater). The reason, according to them, was the aligned orientation of the GNP particles in the injection moulded samples, which prevented them from connecting with each other. This could be also explained by the presence of more PLA and PCL polymers at the surface of the injection-moulded samples, as exhibited by the SEM analysis in the present study (Figure 9a). Through the electrical conductivity measurements using the four-probes method, the electrons are transferred from one external copper probe to the other external probe in a 2D radial pattern [64]. During their trajectory, the electrons cross through the sample’s surface. Subsequently, if the surface is insulating, as in the case of the injection-moulded sample, these electrons are not able to complete their journey. Conversely, the presence of more graphene close to the surface of the 3D-printed samples is highlighted in Figure 9b. In addition, the 3D-printed samples showed stacking of the graphene platelets at the interface of the layers (Figure 10), which explains the electrical conductivity results for these samples in contrast to the injection-moulded samples, which did not possess a similar layered structure.

**Figure 15:**
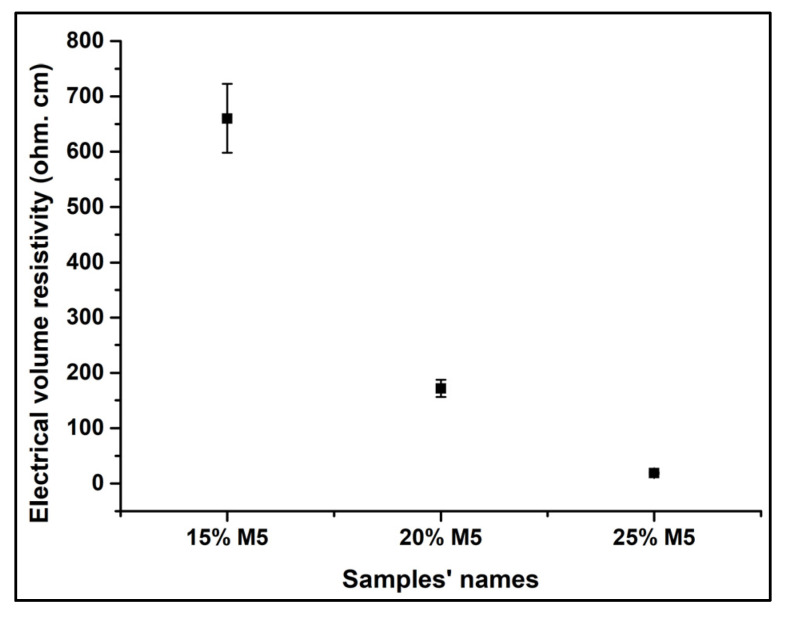
The variation of the electrical volume resistivity of the composites as a function of the percentage of M5 GNP.

The electrical percolation threshold is defined as the minimal percentage of conductive filler required to be added to transform a polymer from an insulating material into a semi-conductive one. For the 3D-printed samples, the 10 wt.% of M5 was under the electrical percolation threshold. However, since the 15 wt.% M5 composite showed an electrical resistance value using the four-probes measurement method, it was considered beyond the electrical percolation threshold. The electrical volume resistivity clearly decreased as the percentage of graphene increased to 20 wt.%. However, a significant decrease in the resistivity was observed when the percentage of graphene reached 25 wt.%. Table 6 and Figure 15 show this decrease in the electrical volume resistivity of the composites with the increase in the percentage of graphene. It has been mentioned that 10 wt.% of graphene is considered to be beyond the rheological percolation threshold, but according to the electrical conductivity results, this composite was electrically insulating. This indicates that the rheological percolated network can be formed at a lower percentage of graphene compared to the electrical percolated network due to the difference in the percolation mechanisms. Rheological percolation requires larger inter-particle distances (tens of nanometers), whereas electrical percolation needs around 5 nm to establish electron hopping. Furthermore, there are three kinds of network in graphene-polymer composites: temporary entangled polymer networks, combined graphene-polymer-graphene networks, and interconnected graphene networks. At low graphene concentrations, graphene sheets exist as individual units because the average distance between them is greater than their own size range. Subsequently, when the graphene concentration reaches the rheological percolation threshold, a polymer-bridged graphene network is constructed in the composites. In this case, the polymer chains attach on the surface of the graphene nanoplatelets and act as bridges to connect the graphene sheets, thus restricting the motion of the polymer chains through pseudo-solid-like behaviour. When the graphene concentration is high enough for these sheets to be in direct contact, a 3D graphene network is constructed to establish the onset of the electrical percolation threshold. Within this network, the free rotation of graphene nanoplatelets is hindered by the adjacent nanoplatelets and the polymer chains are confined to form a high-density interfacial region in the vicinity of these nanoplatelets [65]. Several works dealing with graphene composites have also obtained inferior rheological percolation threshold compared to the electrical percolation threshold [41,66,67,68].

In a work conducted to study the effect of GNP on the physical properties of acrylonitrile-butadiene-styrene nanocomposites, M5 GNP was used. According to the results, for compression-moulded samples containing 15 wt.%, 20 wt.%, and 25 wt.% of M5, the volume electrical resistivity values were approximately 10^6^, 10^4^, and 10^3^ Ω.cm, respectively [69]. In our case, for the same weight percentages of M5 GNP, the obtained resistivity values were 660.2, 171.8, and 18.9 Ω.cm respectively. In another work directed for studying the effect of the graphene nanoplatelets’ morphology on the dispersion and physical properties of polycarbonate composites, and by using M5 GNP, the authors obtained for compression-moulded samples an electrical volume conductivity of 10^−10^ S.cm^−1^ for 15 wt.% of filler, whereas, in our case, the electrical volume conductivity was 1.5 × 10^−3^ S.cm^−1^ [70]. For 20 wt.% of M5, their obtained volume conductivity was 10^−6^ S.cm^−1^, whereas 5.8 × 10^−3^ S.cm^−1^ was achieved in the present study. This indicates that although our printed samples feature some porosity due to the process that is absent in the compression-moulded samples, we obtained higher values of conductivity for the same weight percentages of M5 GNP.

**Figure 16:**
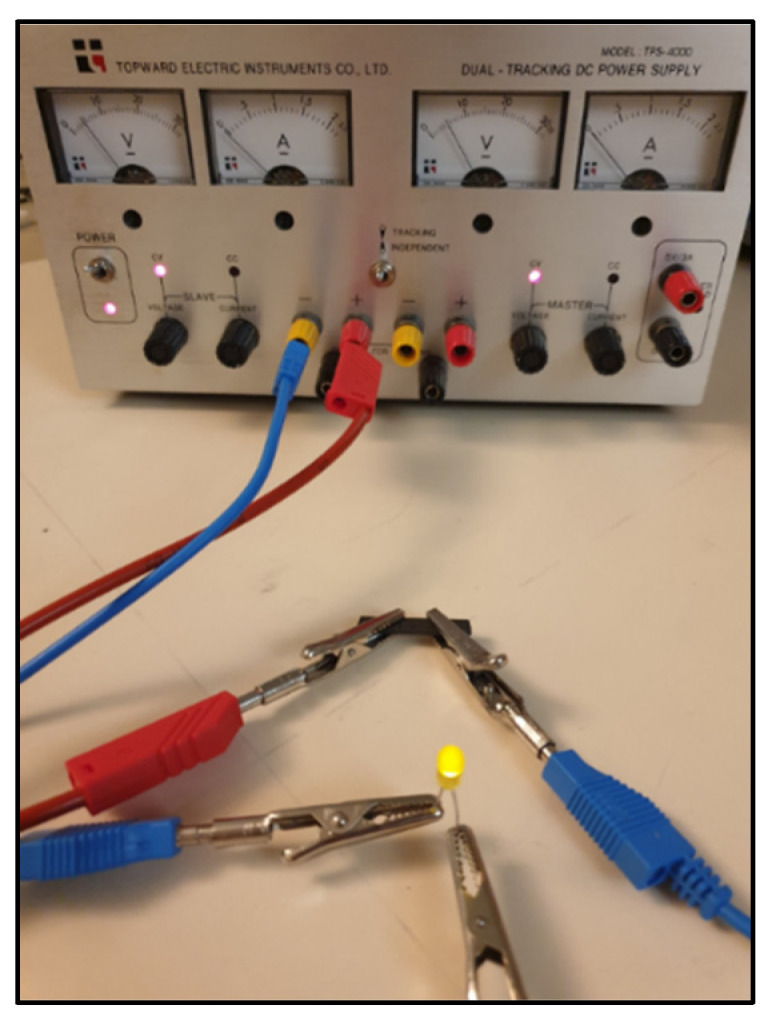
The electrical circuit connected using the 3D-printed PLA/PCL/25 wt.% M5 composite.

To test the applicability of these semi-conductive 3D-printed composites, an electrical circuit was constructed using a voltage generator (voltage of 5 V), a LED, and the 3D-printed sample. When the 3D-printed PLA/PCL/15 wt.% M5 was connected in the circuit, there was no lightening of the LED. However, when the PLA/PCL/20 wt.% M5 and PLA/PCL/25 wt.% M5 were connected instead, the LED lit up. By keeping the same voltage (5 V), the 25 wt.% sample led to more light intensity of the LED due to its lower electrical volume resistivity. The circuit connected using the PLA/PCL/25 wt.% M5 3D-printed composite is shown in Figure 16.

The electrical volume resistivity results of the PLA/PCL/20 wt.% M5 composites printed according to the diagonal pattern (alternating raster angle of +45°/−45°) (Figure 1a), the longitudinal pattern (raster angle of 0°) (Figure 1b), and the transversal pattern (raster angle of 90°) (Figure 1c) are shown in Table 7.

The electrical volume resistivity of the transversal pattern composite was lower than that of the composite printed according to the +45°/−45° raster angle. The composite printed in the longitudinal pattern showed the lowest electrical resistivity. It was reported that the best reinforcement efficiency of GNPs within PA12 matrix was for samples printed according to a 0° raster angle, and this was explained by the fact that in this case, the GNPs were aligned along the stretching direction [39]. It seems that oriented fillers can build conductive pathways more easily, thus leading to lower resistivity. The GNPs are oriented in the direction of stretching; therefore, the raster angle is an important criterion to be taken into consideration when dealing with the electrical conductivity of the material.

## 4. Conclusions

This study shows for the first time the fabrication of semi-conductive PLA/PCL/GNP composites using the FFF technique. The microstructural analysis showed that M5 GNP was selectively localized in PCL rather than PLA; this was ascribed to the lower viscosity and lower melting temperature of PCL, which favoured the localization of the graphene in this polymer during extrusion. This was not in accordance with the wettability prediction, thus revealing that in this case, the kinetic factors defeated the thermodynamic factors. The GNP/PCL nodules were of greater sizes than the pure PCL nodules. Moreover, the thermal tests exhibited that no thermal stability improvement was accomplished in the presence of graphene and this may be ascribed to the aggregation of these nanoparticles in the polymer matrix. The DSC tests revealed that the percentage of crystallinity of PLA increased slightly in the presence of PCL and with the increase in the percentage of graphene. The electrical resistance measurement using the four-probe method indicated that the injection-moulded composites containing 15 wt.%, 20 wt.%, and 25 wt.% were electrically insulators, whereas the 3D-printed composites containing the same percentages of graphene were semi-conductors. In particular, the 20 wt.% and 25 wt.% 3D-printed samples were able to connect an electrical circuit. The reason behind these differences lies in the microstructural differences between both types of samples, which are caused by the process: the injection-moulded samples lack a sufficient number of conductive fillers on their surface, whereas the 3D-printed samples possess graphene platelets on the surface of the printed filaments (during printing on the building platform), whose direction fits to the direction of printing. The presence of a plateau in the storage modulus curves in the low-frequency region suggested the formation of a rheological percolated network that needs a lower graphene percentage compared to the electrical percolated network. The Young’s modulus results were in agreement with the electrical results, and the increase in the graphene percentage led to increases in this property, but a levelling off began from 15 wt.% of GNP. However, probably due to the localization of the graphene sheets on the surface of the printed filaments, which weakens the interfacial adhesion between the layers, the tensile strength decreased with the increase in the percentage of graphene, whose aggregations caused the decrease in the elongation at break of the composites. It is also important to note that the raster angle exerted an impact on the electrical resistance results, according to which the samples printed in the longitudinal direction performed the best for electrical conductivity.

## Figures and Tables

**Table 1:** The surface energy components of water and diiodomethane.

Probe Liquid	${ɣ}_{l}$ mJ.m^−2^	${ɣ}_{l}^{p}$ mJ.m^−2^	${ɣ}_{l}^{d}$ mJ.m^−2^
Water	72.8	51.0	21.8
Diiodomethane	50.8	0.0	50.8

**Table 2:** The solid surface energy, its dispersive and polar components, the test liquids, and the models used for solid surface energy calculations of the PLA, PCL, and GNP (our work and works from the previous studies).

Material	Grade and/or Company	Solid Surface Energy (mJ.m^−2^)	Dispersive Component (mJ.m^−2^)	Polar Component (mJ.m^−2^)	Test Liquids	Model(s)	References
PLA	PLA2003D	39.4 ± 0.6	34.9 ± 1	4.5 ± 0.4	Water, diiodomethane	Owens– Wendt	Present work
	PLA2002D	41.6	30.8	10.8	Water, formamide	Wu’s method	[30]
	PLA4032D	40.6	36.03	4.57	Water, glycerol, diiodomethane	Good and Van Oss	[31]
	PLA3251D	35.5	26.44	9.06	Water, diiodomethane	Wu’s method	[32]
	PLA3D850	43.9 ± 0.7	36.8 ± 0.2	7.1 ± 0.5	Water, diiodomethane, ethylene glycol	Owens–Wendt	[26]
PCL	PCL 6800 Capa Perstorp	35.5 ± 0.2	32.6 ± 0.6	2.9 ± 0.9	Water, diiodomethane	Owens–Wendt	Present work
	PCL6800 Capa Perstorp	42.9 ± 0.5	39.8 ± 0.3	3.1 ± 0.2	Water, diiodomethane, ethylene glycol	Owens–Wendt	[26]
	Sigma Aldrich	40	25	15	Glycerol, water, diiodomethane	Owens–Wendt	[33]
GNP	M5 GNP XG sciences	58.9 ± 0.1	33 ± 0.1	25.9 ± 0.05	Water, diiodomethane	Owens–Wendt	Present work
	Graphene expert Co.	54.8	41.6	13.2	-	-	[34]
	XG sciences	52.6	47.7	4.9	Water, formamide, ethylene glycol, diiodomethane	Owens–Wendt	[35]

**Table 3:** The TGA results of PLA, PCL, their blends, and their composites.

Sample Name	Maximal Degradation Temperature (T_max_) (°C)	Total Mass Loss (%)	Experimental Percentage of Char Yield (at 600 °C)	Theoretical Percentage of Char Yield (at 600 °C)	Onset Temperature (T_onset_) (°C)
PLA	369	100	0	0	350
PCL	417	100	0	0	388
PLA/PCL	366	100	0	0	349
PLA/PCL/10% M5	366	99	7	9	350
PLA/PCL/15% M5	368	91	14	13	354
PLA/PCL/20% M5	366	89	17	18	352
PLA/PCL/25% M5	370	82	23	22	352

**Table 4:** The melting temperatures of PLA and PCL, the crystallization temperature of PLA, and the degree of crystallinity of PLA in the PLA pure polymer, the blend, and the composites.

Sample Name	T_m_ PLA (°C)	T_m_ PCL (°C)	T_c_ PLA (°C)	Degree of Crystallinity of PLA (%)
PLA	149	-	-	0.74
PCL	-	59	-	-
PLA/PCL	150	58	126	1.47
PLA/PCL/10% M5	150	58	125	2.22
PLA/PCL/15% M5	153	59	128	2.35
PLA/PCL/20% M5	151	58	128	2.67
PLA/PCL/25% M5	150	58	123	4.45

**Table 5:** The tensile properties of the 3D-printed PLA, PLA/PCL, and their composites.

Sample name	Young’s Modulus (MPa)	Maximal Tensile Strength (MPa)	Elongation at Break (%)
PLA	3472 ± 151	60.9 ± 1.8	5.1 ± 0.5
PLA/PCL	2748 ± 117	47.4 ± 3.3	12.1 ± 2.2
PLA/PCL/10% M5	3014 ± 67	40.2 ± 1.1	4.7 ± 0.5
PLA/PCL/15% M5	3866 ± 173	39.9 ± 3.4	3.04 ± 0.2
PLA/PCL/20% M5	3744 ± 211	40 ± 2.01	3.2 ± 0.1
PLA/PCL/25% M5	3997 ± 126	34.7 ± 0.8	2.2 ± 0.04

**Table 6:** Electrical volume resistivity of 3D-printed composites with graphene percentages starting from 10 wt.% to 25 wt.% (the raster angle of these samples is +45°/−45°, Figure 1a).

Sample Name	Electrical Volume Resistivity (Ω.cm)
PLA/PCL/10% M5	No results
PLA/PCL/15% M5	660.2 ± 62.3 (9.4%)
PLA/PCL/20% M5	171.8 ± 15.8 (9.2%)
PLA/PCL/25% M5	18.9 ± 1.5 (7.9%)

**Table 7:** The electrical volume resistivity results of the PLA/PCL/20 wt.% M5 composites according to three different patterns by changing the printing raster angles.

Raster Angle	Electrical Volume Resistivity (Ω.cm)
+45°/−45°	171.8 ± 15.8 (9.2%)
0°	106 ± 10.1 (9.5%)
90°	138.4 ± 7.54 (5.4%)

## Data Availability

The data presented in this study are available on request from the corresponding author.
